# Prognostic Significance of the Albumin to Fibrinogen Ratio in Peritoneal Dialysis Patients

**DOI:** 10.3389/fmed.2022.820281

**Published:** 2022-04-28

**Authors:** Wenkai Xia, Meisi Kuang, Chenyu Li, Xiajuan Yao, Yan Chen, Jie Lin, Hong Hu

**Affiliations:** ^1^Department of Nephrology, Jiangyin People's Hospital Affiliated to Nantong University, Jiangyin, China; ^2^Nephrologisches Zentrum, Medizinische Klinik und Poliklinik IV, Klinikum der Universität München, Ludwig-Maximilians-University Munich, Munich, Germany

**Keywords:** albumin, fibrinogen, peritoneal dialysis (PD), prognosis, biomarker

## Abstract

**Background:**

Albumin to fibrinogen ratio (AFR) is a demonstrated predictor of mortality in various diseases. The aim of this study was to evaluate the prognostic value of AFR to predict mortality in peritoneal dialysis (PD) patients.

**Methods:**

We retrospectively analyzed 212 incident PD patients from January 2010 to December 2017 and followed them until December 2019. We used receiver operating curve (ROC) analysis to determine the optimal cut-off point for AFR at baseline to predict overall and cardiovascular mortality during the follow-up period. Kaplan-Meier curve and Cox regression analysis were applied to evaluate the association between AFR and all-cause and cardiovascular mortality.

**Results:**

The optimal threshold for AFR to predict mortality was 8.48. A low AFR was strongly correlated with worse all-cause and cardiovascular mortality in PD patients. Multivariate analysis revealed that elevated AFR was an independent marker predicting reduced all-cause and cardiovascular mortality (HR 2.41, 95% CI 1.11–5.22, *P* = 0.026; and HR 2.18, 95% CI 1.21–3.95, *P* = 0.010, respectively).

**Conclusions:**

Patients with a high AFR had reduced all-cause and cardiovascular mortality. AFR is a potential prognostic biomarker in PD patients.

## Introduction

Chronic kidney disease (CKD) is a major public health concern worldwide. Peritoneal dialysis (PD) has emerged as one of the most commonly used and efficient treatment options for CKD. However, patients with CKD still have an excessive risk of all-cause mortality and particularly cardiovascular (CV) events (1). Effective interventions must therefore be well-recognized to support CKD prevention and management.

Malnutrition is highly prevalent in CKD patients, especially among patients on dialysis, which leads to low albumin levels (2). However, recent studies have proposed that hypoalbuminemia negatively correlated with patients' prognosis could be attributed more to inflammation than to malnutrition (3–6). Furthermore, fibrinogen has been found to go beyond its conventional role in coagulation and is a readily available predictor of microinflammation (7, 8). An increased fibrinogen level has been shown to predict the development of CV events and mortality in the general population and in patients undergoing PD (9, 10).

In recent decades, systemic inflammation has been considered a hallmark feature of CKD, promoting CKD progression as well as enhancing cardiovascular mortality. As a novel inflammation-based indicator, the albumin to fibrinogen ratio (AFR) has gained prognostic value in various cancers (11, 12) and other diseases such as acute pancreatitis and rheumatoid arthritis (13, 14). However, there has been no research investigating the predictive value of AFR in PD patients. In this retrospective study, we aimed to evaluate the association of AFR with all-cause and cardiovascular mortality in incident PD patients.

## Patients and Methods

### Patients and Data Collection

**Figure 1:**
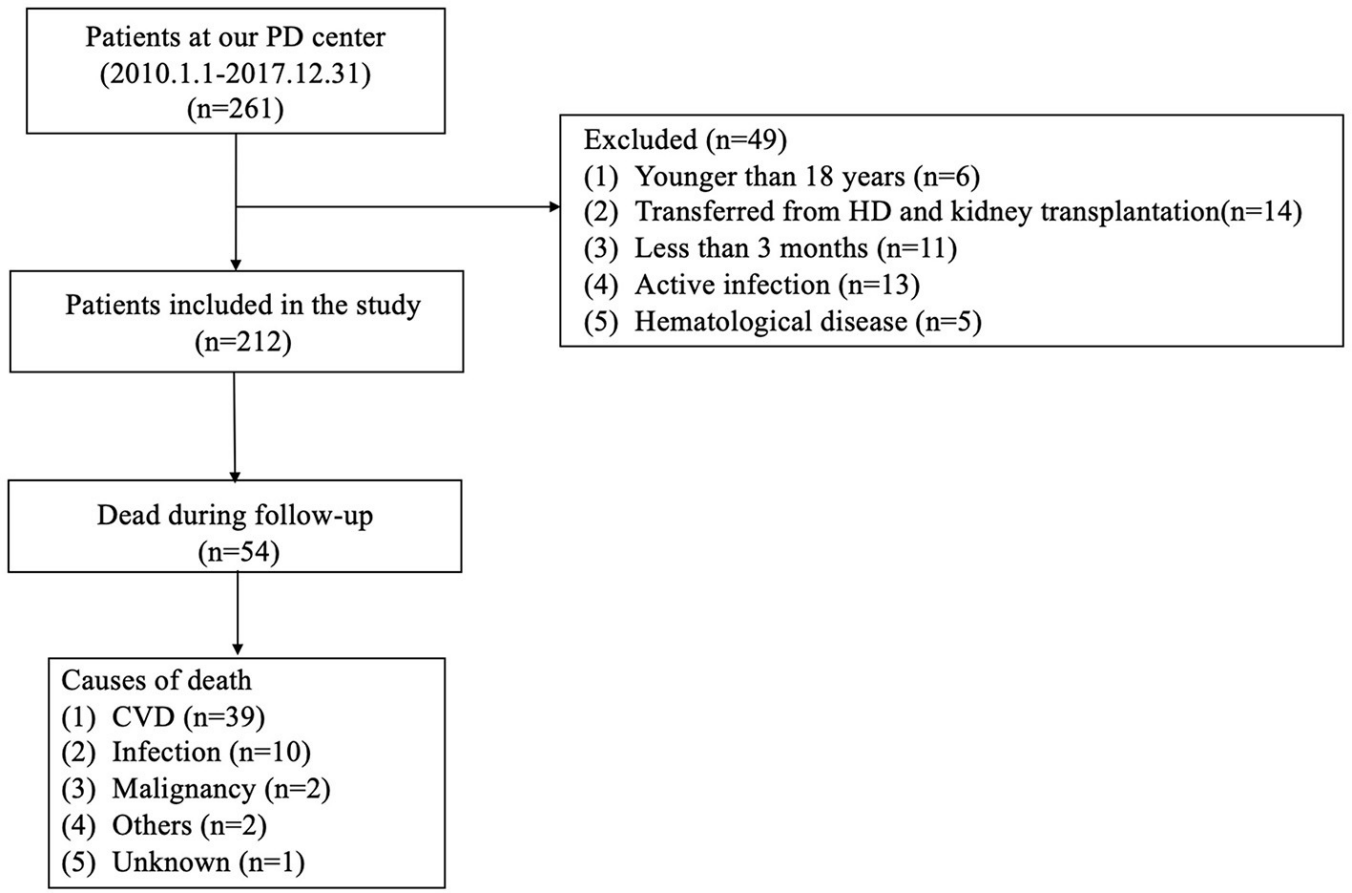
Enrollment flowchart for analysis. PD, peritoneal dialysis; HD, hemodialysis; CVD, cardiovascular disease.

This was a retrospective observational cohort study. Medical records of all incident PD patients between January 2010 and December 2017 were collected at the Jiangyin People's Hospital Affiliated to Nantong University and retrospectively analyzed in this study. Patients who were aged ≥18 years old and received PD treatment for at least three consecutive months were included. The exclusion criteria were as follows: (1) patients with a history of previous hemodialysis (HD) or kidney transplantation; (2) active infection, hyperpyrexia, and hematological disease within 3 months of PD treatment; (3) patients lost to follow-up (Figure 1).

The enrolled patients received the conventional PD solutions (Dianeal 1.5%, 2.5%, or 4.25% dextrose; Baxter Healthcare, Guangzhou, China), with 3–5 exchanges per day. All patients were followed up for a maximum of 5 years, from the initiation of treatment to death or the end point time. Baseline demographic data were collected at the first 1–3 months after the initiation of PD treatment, and included age, sex, history of hypertension, cardiovascular disease (CVD) and diabetes, body mass index (BMI), neutrophils, leukocytes, serum albumin, globulin, fibrinogen, creatinine, urea nitrogen, and D-dimer. Each patient was regularly followed up at least quarterly, with a physical examination and laboratory testing. Informed consent was waived by the Ethics Committee due to the retrospective and non-interventional nature of the study.

### Study Definitions

The NLR was obtained as the ratio of the neutrophil count to the lymphocyte count. The AFR was obtained by dividing the serum albumin level by the plasma fibrinogen level. The primary outcome was all-cause mortality. The secondary outcome was cardiovascular mortality, which was defined as death caused by congestive heart failure, cardiac arrhythmia, acute myocardial infarction, angioplasty, coronary artery bypass, or stroke. CVD was defined as a history of myocardial infarction, coronary artery bypass, heart failure, atherosclerotic heart disease, or stroke. Patients who had a previous history of type 1 and 2 diabetic mellitus and/or who reported current use of insulin or oral hypoglycemic agents were considered to have diabetes mellitus. Hypertension was defined as taking antihypertensive agents or 2 separate blood pressure measurements ≥140/90 mmHg.

### Statistical Analysis

Continuous variables are presented as mean ± standard deviation (SD) or median (interquartile range) and categorical data are expressed as number or percentage. The optimal cut-off value of AFR was determined by receiver operating curve (ROC) analysis, and patients were then divided into two groups according to the optimal threshold. The chi-squared, Mann-Whitney *U*, or Kruskal-Wallis test was used to test the characteristic difference in categorical or continuous factors between groups. Multivariate Cox regression analysis with mortality as outcome was performed to calculate the hazard ratios (HRs). Statistical analysis was performed with SPSS 20.0 software (SPSS Inc., IBM, USA). *P* < 0.05 was considered statistically significant.

## Results

### Patients' Baseline Characteristics

A total of 212 patients undergoing PD were finally enrolled in this study. The mean age was 50 ± 14 years, 124 (58.5%) were male, 121 (57.1%) had hypertension, 41 (19.3%) had diabetes mellitus, and 15 (7.1%) had a history of CVD. The optimal AFR cut-off levels based on overall survival (OS) and cardiac-specific survival, respectively, were determined to be 10.67 (50.0% sensitivity and 79.6% specificity) and 8.48 (76.9% sensitivity and 63.5% specificity) by ROC curve analysis (Figure 1). Patients were subsequently divided into two groups according to the optimal cut-off value calculated by the endpoint based on OS. Patients in the low AFR group were older and had higher rates of hypertension and diabetes as well as high levels of NLR, globulin, and fibrinogen, but lower levels of albumin. The clinical baseline characteristics of all patients according to low vs. high AFR are listed in Table 1.

**Table 1:** Baseline characteristics according to AFR.

**Variables**	**AFR < 10.67** ***n* = 122**	**AFR ≥ 10.67** ***n* = 90**	***P***
Age, y	53 ± 14	46 ± 13	<0.001
Male, (*n*, %)	73 (59.8)	51 (56.7)	0.643
BMI	21.7 ± 2.1	21.8 ± 2.2	0.297
Total Kt/V	1.9 (1.7–2.3)	2.1 (1.8–2.3)	0.071
**Laboratory data**			
Hb (g/L)	95.6 ± 21.8	97.8 ± 16.6	0.420
NLR	4.7 ± 3.5	3.3 ± 2.4	0.002
Albumin (g/L)	32.8 ± 4.0	36.5 ± 3.6	<0.001
Globulin (g/L)	24.6 ± 5.2	22.8 ± 5.0	0.014
BUN (mmol/L)	17.8 ± 6.2	18.3 ± 7.0	0.559
Creatinine (μmol/L)	806.0 (656.2–1,094.1)	875.5 (712.3–1,053.6)	0.120
Fibrinogen (g/L)	4.2 ± 0.9	2.8 ± 0.4	<0.001
D-dimer (mg/L)	1.1 (0.5–1.7)	0.7 (0.2–1.5)	0.785
**Comorbid conditions (** ***n*** **, %)**			
Hypertension	84 (68.9)	37 (41.1)	<0.001
Diabetes mellitus	33 (27.0)	8 (8.9)	0.001
CVD	12 (9.8)	3 (3.3)	0.102
SBP (mmHg)	148.3 ± 23.6	148.5 ± 20.2	0.948
DBP (mmHg)	87.9 ± 16.1	91.9 ± 13.5	0.057

*Values are described as mean ± standard deviations, median and interquartile range, or number (percentage) as appropriate*.

*BMI, body mass index; Hb, hemoglobin; NLR, neutrophil to lymphocyte ratio; AFR, albumin to fibrinogen ratio; BUN, blood urea nitrogen; CVD, cardiovascular disease; SBP, systolic blood pressure; DBP, diastolic blood pressure*.

### Prognostic Value of AFR for All-Cause and Cardiovascular Mortality

**Figure 2:**
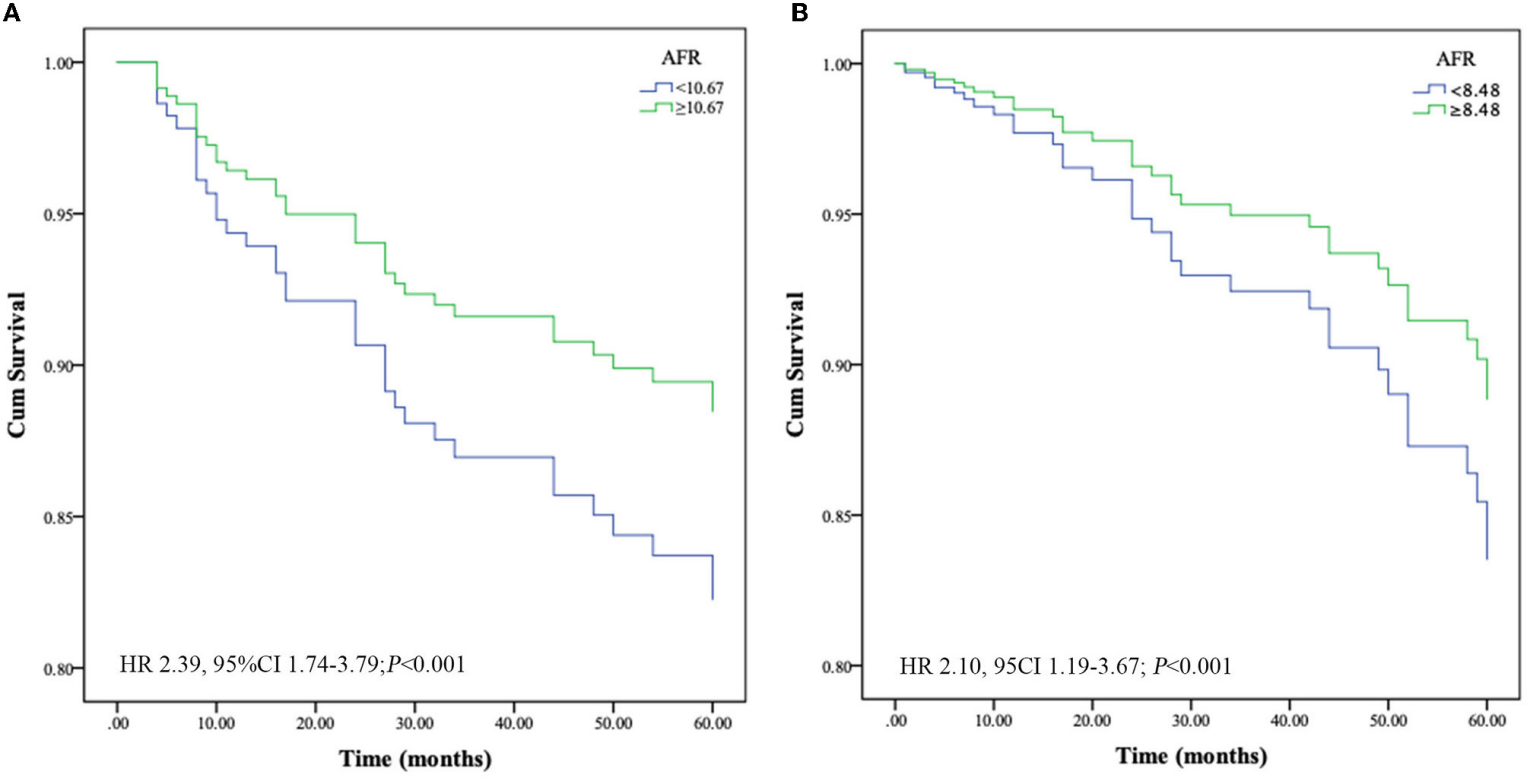
Optimal thresholds for fibrinogen, albumin, AFR, and NLR were applied with ROC curves for **(A)** all-cause mortality and **(B)** cardiovascular mortality. NLR, neutrophil to lymphocyte ratio; AFR, albumin to fibrinogen ratio; AUC, area under curve; CI, confidence interval.

At the end of follow-up, we recorded 54 deaths, of which 39 (72.2%) were due to cardiovascular mortality during the follow-up period. Kaplan-Meier survival analysis and log-rank testing were used to determine the association between AFR and all-cause and cardiovascular mortality. Our results demonstrated that a lower AFR was significantly associated with decreased OS (Figure 2A). Similarly, patients with lower AFRs also had an increased risk for cardiovascular mortality compared with patients with higher AFRs (Figure 2B). Furthermore, multivariate Cox regression analysis indicated that a low AFR was independently associated with reduced overall survival (HR 2.39, 95% CI 1.74–3.79, *P* < 0.001). In addition, age (HR 1.04, 95% CI 1.00–1.08, *P* = 0.034), a history of hypertension (HR 2.76, 95% CI 1.05–7.26, *P* = 0.040), and NLR (HR 1.19, 95% CI 1.04–1.22, *P* = 0.002) were independent indicators of all-cause mortality (Table 2). Similarly, results from multivariate analysis revealed that a low AFR was associated with increased cardiovascular mortality (HR 2.10, 95% CI 1.19–3.67, *P* < 0.001). Age (HR 1.07, 95% CI 1.03–1.11, *P* < 0.001), a history of diabetes mellitus (HR 2.38, 95% CI 1.09–5.21, *P* = 0.030), and NLR (HR 1.11, 95% CI 1.03–1.20, *P* = 0.002) were independent risk factors for cardiovascular mortality in PD patients (Table 2).

**Table 2:** Independent factors correlated with all-cause and cardiovascular mortality.

	**Hazard ratio**	**95% confidence interval**	***P*-value**
**All-cause mortality**			
Age	1.04	1.00–1.08	0.034
Hypertension	2.76	1.05–7.26	0.040
NLR	1.19	1.10–1.29	<0.001
AFR (<10.67)	2.39	1.74–3.79	<0.001
**Cardiovascular mortality**			
Age	1.07	1.03–1.11	<0.001
Diabetes mellitus	2.38	1.09–5.21	0.030
NLR	1.13	1.04–1.22	0.002
AFR (<8.48)	2.10	1.19–3.67	<0.001

*NLR, neutrophil to lymphocyte ratio; AFR, albumin to fibrinogen ratio*.

**Figure 3:**
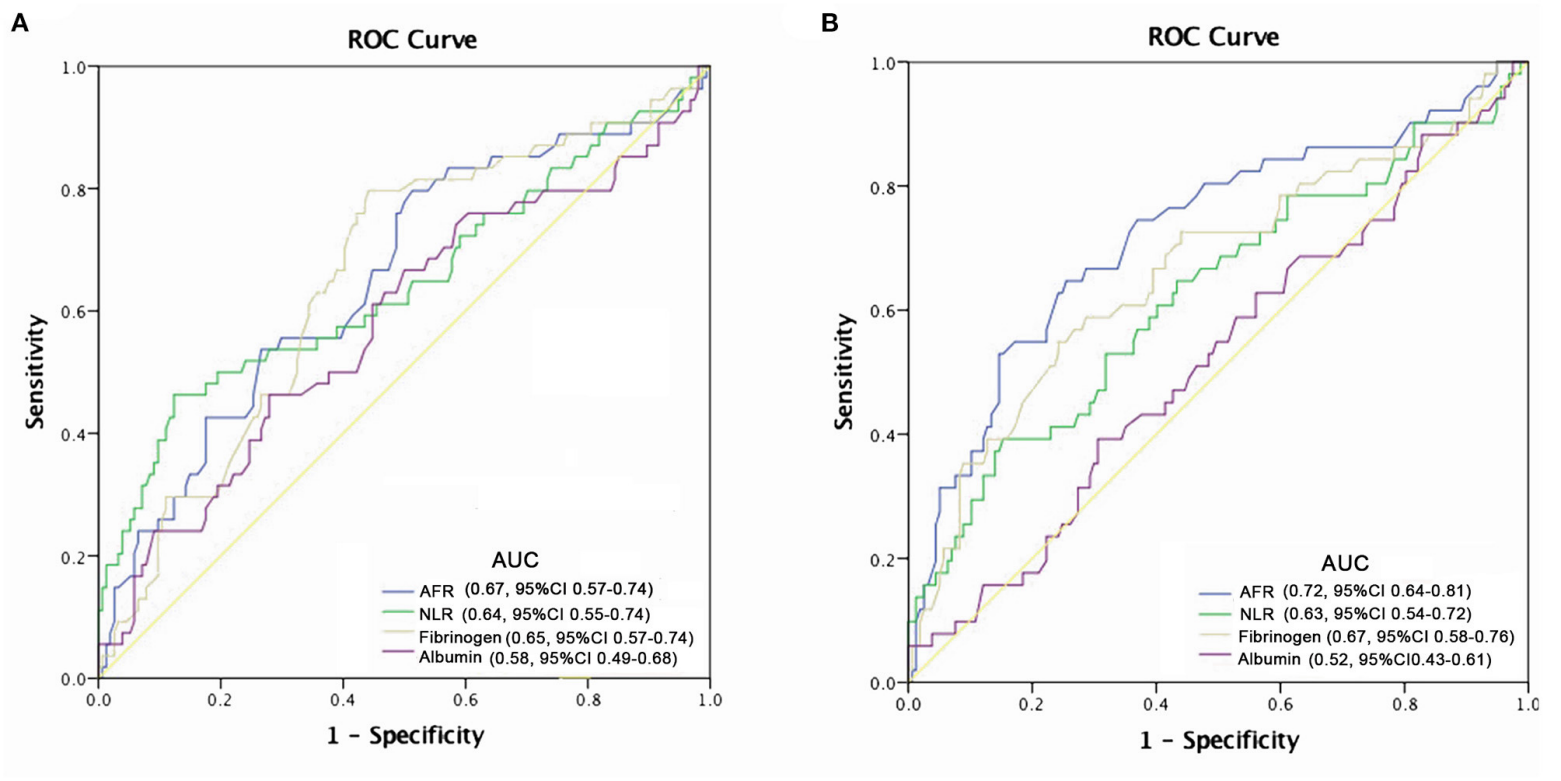
Crude analysis comparing all-cause and cardiovascular mortality between AFR groups. Cumulative mortality curves for **(A)** all-cause mortality and **(B)** cardiovascular mortality. PD, peritoneal dialysis; AFR, albumin to fibrinogen ratio; HR, hazard ratio; CI, confidence interval.

The predictive power of AFR and other indicators are depicted in Figure 2. Compared with albumin, fibrinogen, and NLR, AFR exhibited improved power for predicting all-cause and cardiovascular mortality in PD patients (Figure 3).

## Discussion

In this retrospective study of PD patients, we explored the prognostic performance of AFR in predicting their overall and cardiovascular mortality. An elevated AFR was most significantly associated with reduced all-cause mortality as well as reduced cardiovascular mortality. In addition, the combination of albumin and fibrinogen with AFR tended to better predict risk for all-cause and cardiovascular mortality than the individual markers alone.

Serum albumin has been used to reflect malnourished status (15). Hypoalbuminemia is reported to be a key characteristic of protein-energy wasting, which is an important risk factor for higher mortality and is highly prevalent in CKD and PD patients (16, 17). However, emerging evidence has demonstrated that serum albumin is inversely correlated with proinflammatory cytokines in PD patients and that inflammatory cytokines such as TNF-α and IL-6 may suppress albumin synthesis, suggesting hypoalbuminemia is attributable more to systemic inflammation than to malnutrition in patients undergoing PD (18, 19). Taken together, hypoalbuminemia is associated with increased mortality in PD patients and is attributed in part to inflammation.

Previous studies revealed that plasma fibrinogen is always lower in PD patients than in hemodialysis (HD) patients. The results demonstrated that the loss of albumin in the peritoneal dialysate leads to the accumulation of free fatty acids in the blood, which stimulates fibrinogen synthesis for the liver (20, 21). On the other hand, long-term and continuous exposures to glucose-based dialysate results in severe metabolic syndrome, leading to defective endothelial function, aggravated inflammation, and prothrombotic tendency (22). Therefore, patients who receive PD treatment exhibit a more prothrombotic profile. Several studies have investigated whether elevated plasma fibrinogen is a risk factor for all-cause and cardiovascular mortality in CKD and dialysis patients, but have yielded conflicting results. Indeed, a large retrospective study demonstrated that plasma fibrinogen levels are not a strong risk factor for cardiovascular mortality in individuals with CKD (23), indicating a complex relationship between fibrinogen and mortality in this patient population. In contrast, studies conducted on exclusively HD or PD patients reported a significant association of elevated fibrinogen levels with all-cause and cardiovascular mortality (10, 24). The reasons for these contradictory results regarding fibrinogen and cardiovascular mortality in non-dialyzed and dialysis patients remain unknown. Interestingly, plasma fibrinogen was found to be glycated and later oxidized through post-translational modifications, which may be particularly prevalent in diabetic patients and associated with increased clot density, which drives cardiovascular mortality.

Recent evidence supports the prognostic value of AFR, with the majority of studies focused on various types of cancer. Pretreatment AFR can act as a promising prognostic indicator in patients with lung cancer (11), esophageal squamous cell carcinoma (25), and gastric cancer (26). Furthermore, low AFR can improve diagnostic efficiency in cervical cancer (27). Importantly, our results confirmed the independent relationship between a low AFR and overall and cardiovascular mortality in PD patients. We found that increased AFR was independently correlated with reduced all-cause and cardiovascular mortality in PD patients. The optimal cut-off point of 8.48 for AFR was the best predictor based on hazard ratio, achieving the best sensitivity and specificity. In addition, ROC curve analysis from our study revealed that the AUC of AFR was larger than that of albumin and fibrinogen alone, suggesting that AFR could amplify the sensitivity and specificity of predicting the survival of patients undergoing PD. In addition, the prevalence of hypertension was increased in the low AFR group and hypertension was found to contribute to all-cause mortality. In multivariate Cox regression, TG, HDL-C, and NLR were associated with all-cause mortality in PD patients, consistent with previous studies (28, 29). Furthermore, age and higher NLR, the well-known risk factors for cardiovascular events, were also demonstrated to be independently correlated with cardiovascular mortality.

Several limitations should be acknowledged. First, this was a single-center retrospective study with a modest sample size, which may result in inherent biases. Second, we used only the baseline of AFR in the analysis, without considering the impact of variations in the AFR during the follow-up period. Third, we had no available data on CRP, insulin resistance, and inflammatory parameters such as tumor necrosis factor and IL-6, since they were not routinely measured. Finally, a prospective cohort with multi-center designs, a large sample size, and longer follow-up length are warranted to verify our findings.

## Conclusion

In conclusion, our study demonstrates that AFR is an independent predictor of overall and cardiovascular mortality in PD patients. Since the assessment of AFR is economical, accessible, easy to measure, and has standard criteria worldwide, AFR is a promising new marker with which to identify high-risk PD patients.

## Data Availability Statement

The raw data supporting the conclusions of this article will be made available by the authors, without undue reservation.

## Ethics Statement

The studies involving human participants were reviewed and approved by the Medical Ethics Committee of Jiangyin People's Hospital Affiliated to Nantong University. Written informed consent for participation was not required for this study in accordance with the national legislation and the institutional requirements.

## Author Contributions

WX and HH: study design. YC, JL, and XY: data collection and analysis. WX and MK: manuscript drafting. CL and HH: editing and revising. All authors have approved the submitted version and agreed to be accountable for their own contributions.

## Funding

This study was supported by a grant from the Scientific Research Project of the Wuxi Health Committee (Q201754). The funding body had no role in the design of the study, the collection, analysis, interpretation of the data, and the writing of the manuscript.

## Conflict of Interest

The authors declare that the research was conducted in the absence of any commercial or financial relationships that could be construed as a potential conflict of interest.

## Publisher's Note

All claims expressed in this article are solely those of the authors and do not necessarily represent those of their affiliated organizations, or those of the publisher, the editors and the reviewers. Any product that may be evaluated in this article, or claim that may be made by its manufacturer, is not guaranteed or endorsed by the publisher.
